# A case of intraoperative detection of a central venous catheter in azygos vein arch during esophageal cancer surgery

**DOI:** 10.1186/s40792-024-02055-w

**Published:** 2024-11-11

**Authors:** Katsuhiko Murakawa, Koichi Ono, Yoshiyuki Yamamura

**Affiliations:** Department of Surgery, Obihiro Kosei Hospital, West 14 South 10, Obihiro, 080-0024 Japan

**Keywords:** Central venous catheter, Azygos vein arch, Esophageal cancer

## Abstract

**Background:**

Central venous catheter (CVC) is often used in the perioperative management of esophageal cancer. The position of the CVC tip has been reported to shift with body positioning and, although infrequent, may traverse into the azygos vein arch. Herein, we describe a case where a migrated CVC tip in the azygous vein arch was identified during esophageal cancer surgery, preventing CVC dissection concurrent with azygous vein arch resection.

**Case presentation:**

A 65-year-old man was diagnosed with advanced esophageal cancer and was referred to our department for surgery after undergoing neoadjuvant chemotherapy. He underwent robot-assisted subtotal esophagectomy, followed by gastric conduit reconstruction via the posterior sternal route. Thoracic manipulation was performed with the patient in the prone position. During the surgery, a foreign body was found in the azygos vein arch, indicating that a central venous catheter had inadvertently entered the azygos vein arch. The catheter was retracted by 5 cm, and after confirming that no catheter remained in the azygos arch, the azygos vein arch was separated using an autosuture device.

**Conclusions:**

Central venous catheter migration can occur in a various vessels. During prone esophageal cancer surgery, elevating the right upper extremity may alter the catheter tip’s position from its the preoperative position. CVC amputation should be observed because the azygos vein arch is often amputated to facilitate upper mediastinal dissection during esophageal cancer surgery.

## Introduction

Central venous catheter (CVC) is often used in the perioperative management of esophageal cancer. The position of the CVC tip has been reported to shift with body positioning and, although infrequent, may traverse into the azygos vein arch [1]. CVC amputation should be observed because the azygos vein arch is often amputated to facilitate upper mediastinal dissection during esophageal cancer surgery. Herein, we describe a case where a migrated CVC tip in the azygous vein arch was identified during esophageal cancer surgery, preventing CVC dissection concurrent with azygous vein arch resection.

## Case report

**Fig. 1:**
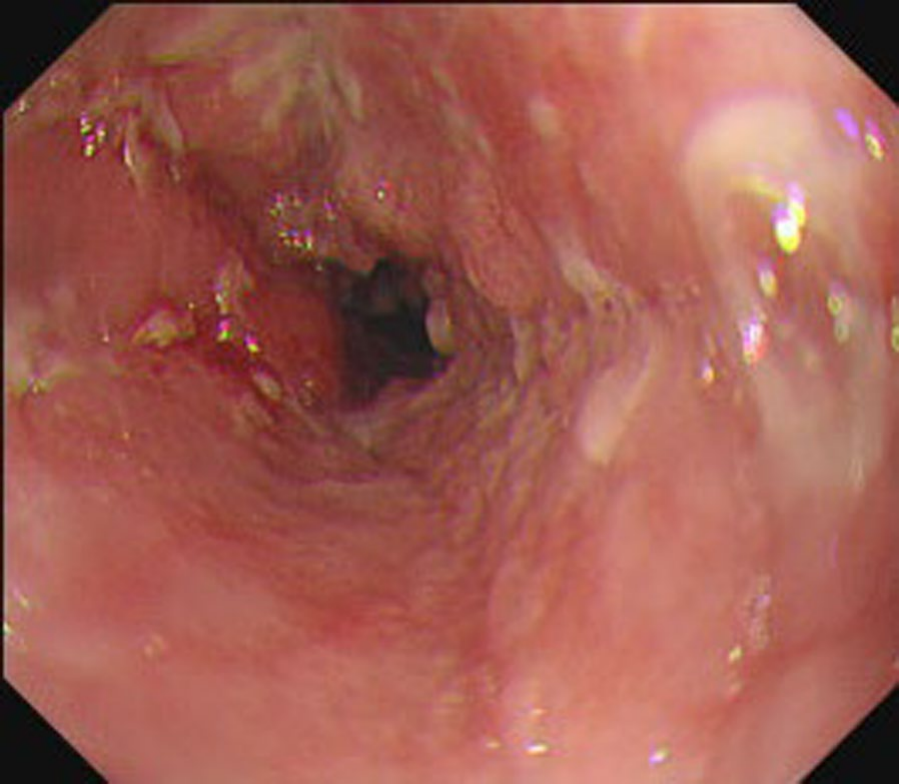
Advanced cancer of the thoracic esophagus was observed

**Fig. 2:**
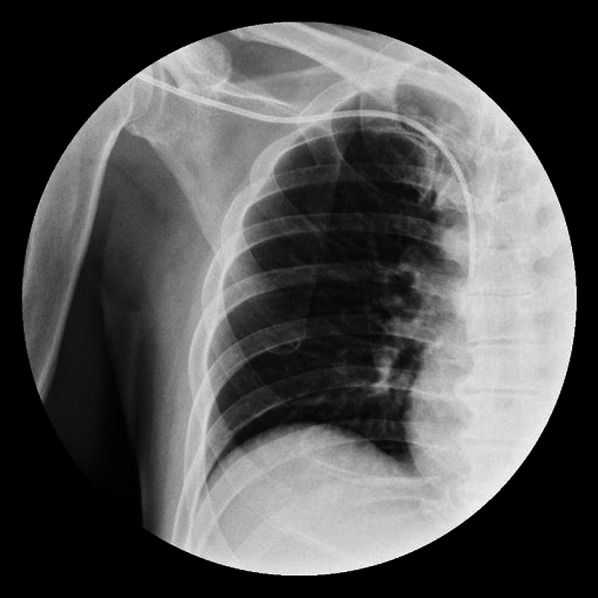
The CVC tip is located in the superior vena cava at the level of the tracheal bifurcation

**Fig. 3:**
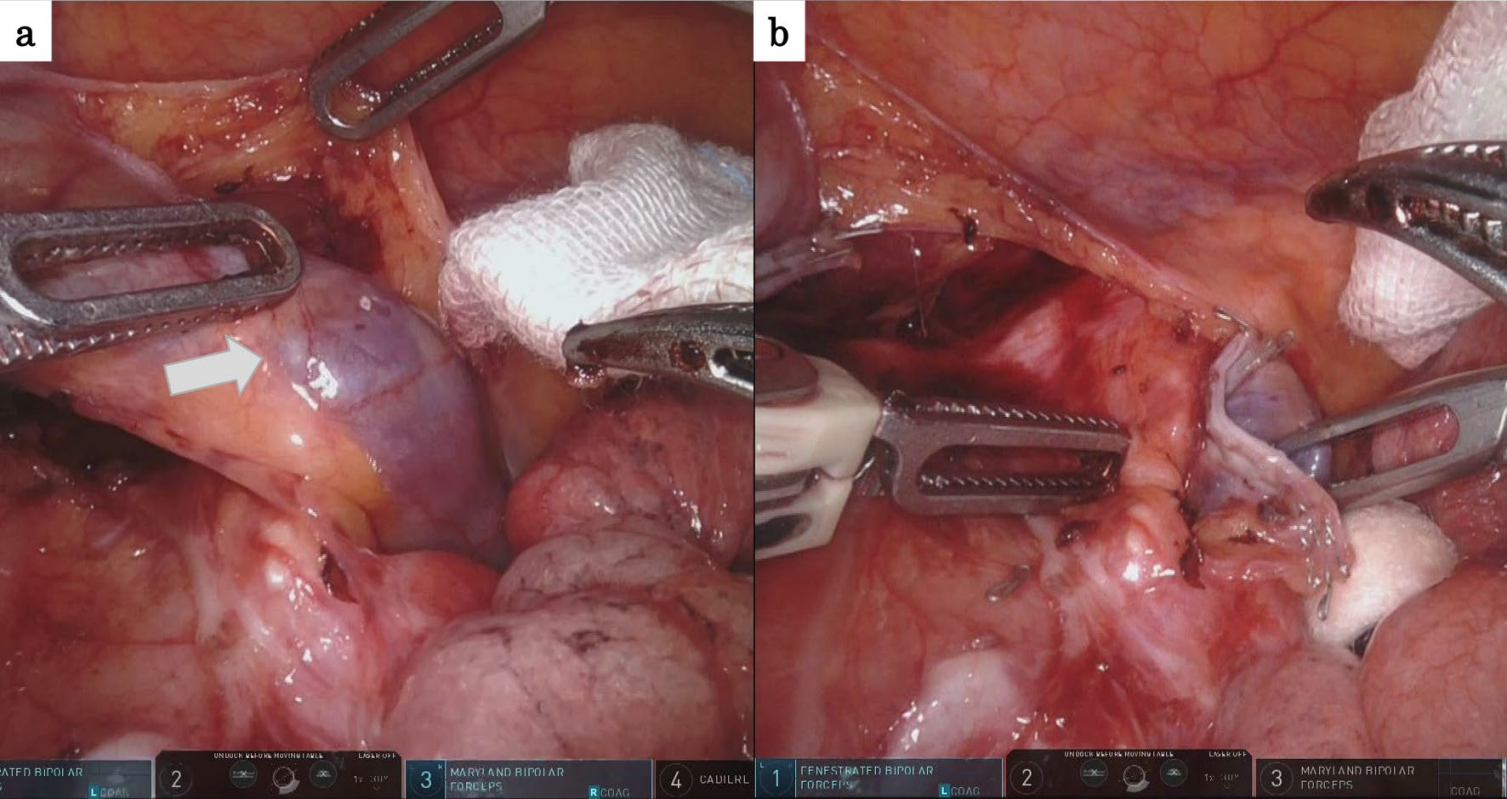
A foreign body is seen in the azygos vein arch (**a**). No CVC is observed at the cut line (**b**)

A 65-year-old man with dysphagia was referred to a previous clinic. He had no prior medical history and was diagnosed with advanced esophageal cancer. He was subsequently referred to our department for surgery after undergoing three courses of docetaxel plus 5-fluorouracil and cisplatin induction chemotherapy as the preoperative treatment plan. Upper gastrointestinal endoscopy revealed a type 3 tumor in the thoracic esophagus, and biopsy results lead to a diagnosis of squamous cell carcinoma (Fig. 1). Esophagography showed a 3-cm type 3 tumor in the upper thoracic esophagus. Computed tomography revealed wall thickening in the upper esophagus; however, no lymphadenopathy or distant metastasis was detected. The disease was categorized as cT3N0M0, Stage II according to the Japanese Classification of Esophageal Cancer 12th Edition, and the tumor achieved partial response to preoperative chemotherapy [2]. The day prior to surgery, a CVC was inserted thorough the right subclavian vein under radiographic fluoroscopy and placed in the superior vena cava (Fig. 2). The patient underwent robot-assisted subtotal esophagectomy using the da Vinci Xi surgical system (Intuitive Surgical Inc., CA, USA) and subsequent reconstruction of the gastric conduit through the retrosternal route. During thoracoscopic phase, he was placed in the prone position, with the right arm abducted. Four 8-mm da Vinci ports and one 12-mm assistant port were inserted. Following mid and lower mediastinal dissection, we exposed the azygos vein arch. At that moment, a foreign body was identified in the azygos vein arch, and we were convinced that it was the CVC tip (Fig. 3a). The CVC was retracted by 5 cm by the anesthesiologist, and after confirming that the tip was no longer in the azygos vein arch, the azygos vein arch was separated using an autosuture device (Fig. 3b). Subsequently, an upper mediastinal dissection was performed, and the thoracic manipulation was completed. Sub-total esophagectomy, three-region lymphadenectomy, and the reconstruction were performed. The surgical duration was 8 h and 26 min, and the amount of blood loss was 52 g. Enteral nutrition was started on postoperative day 1, and the CVC was removed on postoperative day 4. The patient’s postoperative course was uneventful, and he was discharged on postoperative day 21.

## Discussion

**Fig. 4:**
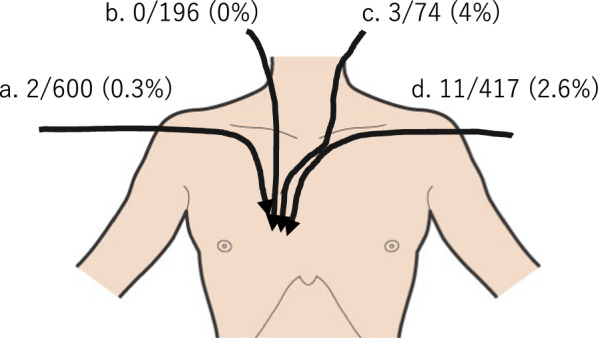
Bankier et al. demonstrated absolute numbers and frequencies of the catheter malpositions in the azygos arch. **a** Right subclavian vein. **b** Right jugular vein. **c** Left jugular vein. **d** Left subclavian vein

CVCs are often utilized for intraoperative esophageal cancer management or postoperative nutrition. They are typically inserted preoperatively, and the location of the CVC tip is confirmed using X-rays. The migration rate of the central venous catheters into the azygos vein arch is reported to be approximately 1.2% and is more common when inserted from the left side compared with the right side (Fig. 4) [3]. And the incidence of migration into the azygos vein arch when inserted via the right subclavian vein, as in our case, is reported to be 0.3%. This migration can lead to complications including venipuncture. Prabaharan et al. reported a case where a CVC inserted from the right subclavian vein into the left subclavian vein SVC migrated to the SVC after extubation [4]. Talari et al. reported repeat migration of a peripherally inserted central catheter (PICC) tip into the azygos vein arch [5]. Forauer et al. observed that hand abduction or adduction causes the PICC tip to move an average of 21 mm and a maximum of 53 mm [1]. External conditions can influence the position of the CVC tip, implying that it may not consistently remain in the same position. And Zhang has pointed out that soft silicone PICCs are prone to tip migration [6].

Currently, esophageal cancer surgery is often performed in the left lateral recumbent or prone position, and the right upper extremity is often abducted and elevated [7]. Conversely, CVC insertion occurs in the supine position and confirmed by chest X-ray. This necessitates recognition that the CVC tip position may change during esophageal cancer surgery performed in the left lateral recumbent or prone positions.

During esophageal cancer surgery, the azygos vein arch is often transected to facilitate the approach to the left recurrent nerve lymph nodes. In the present case, identification of the CVC tip in the azygos vein arch prevented catheter amputation by safely removing it. Reportedly, a case exists where the CVC tip, having migrated into the azygos vein arch, required amputation [6]. Amputating a CVC can pose greater harm to the patient, potentially leading to the conversion of thoracoscopic surgery to an open thoracotomy or necessitating revision surgery, depending on the timing of the diagnosis.

Recognizing intraoperatively a CVC tip that has migrated into the azygos vein arch is challenging. Surgical options to prevent CVC dissection include preservation of the azygos vein arch through right thoracic and cervical mediastinoscopy approaches without dissection of the azygos vein arch, both of which are challenging [8, 9]. Although confirming the tip position using radiograph after CVC placement is essential, many factors can influence the position of the CVC tip during esophageal cancer surgery, such as positional changes, intraoperative changes in intrathoracic pressure, and intraoperative manipulations. To prevent cutting the catheter within the azygos arch, it is important to avoid insertion from the left side, where the catheter is more likely to enter the azygos vein arch. Most importantly, the azygos vein arch should be thoroughly examined to ensure the absence of a CVC before dissecting the azygos vein arch.

## Conclusion

Central venous catheter migration can occur in a various vessels. During prone esophageal cancer surgery, elevating the right upper extremity may alter the catheter tip’s position from its preoperative position. CVC tip’s position should be observed because the azygos vein arch is often amputated to facilitate upper mediastinal dissection during esophageal cancer surgery.

## Data Availability

Not applicable.

## Abbreviations

CVC: Central venous catheter
PICC: Peripherally inserted central catheter
